# Correction to: LILRB2-containing small extracellular vesicles from glioblastoma promote tumor progression by promoting the formation and expansion of myeloid-derived suppressor cells

**DOI:** 10.1007/s00262-023-03426-2

**Published:** 2023-04-17

**Authors:** Peitao Wu, Yuhang Guo, Li Xiao, Jiaqi Yuan, Chao Tang, Jun Dong, Zhiyuan Qian

**Affiliations:** 1 Department of Neurosurgery, The Second Affiliated Hospital of Soochow University, Soochow, 215000 People’s Republic of China; 2 Department of Neurosurgery, The First Affiliated Hospital of Wenzhou Medical University, Nanbaixiang, Wenzhou, China; 3 Department of Neurosurgery, Huashan Hospital, Shanghai, China

**Correction to: Cancer Immunology, Immunotherapy** 10.1007/s00262-023-03395-6

The original version of this article unfortunately contained a mistake. The corrected details are given below for your reading.

Equal contributor statement was missing. Equal contributor statement should be "Yuhang Guo is the co-first author".

The statistical result in Fig. 2B has some mistakes.

The statistical analysis was not markered in Fig. 3A, C and F, and the statistical analysis result of Fig. 3B was misplaced.

The corrected Figs. 2 and 3 are given in the following page.

**Fig. 2:**
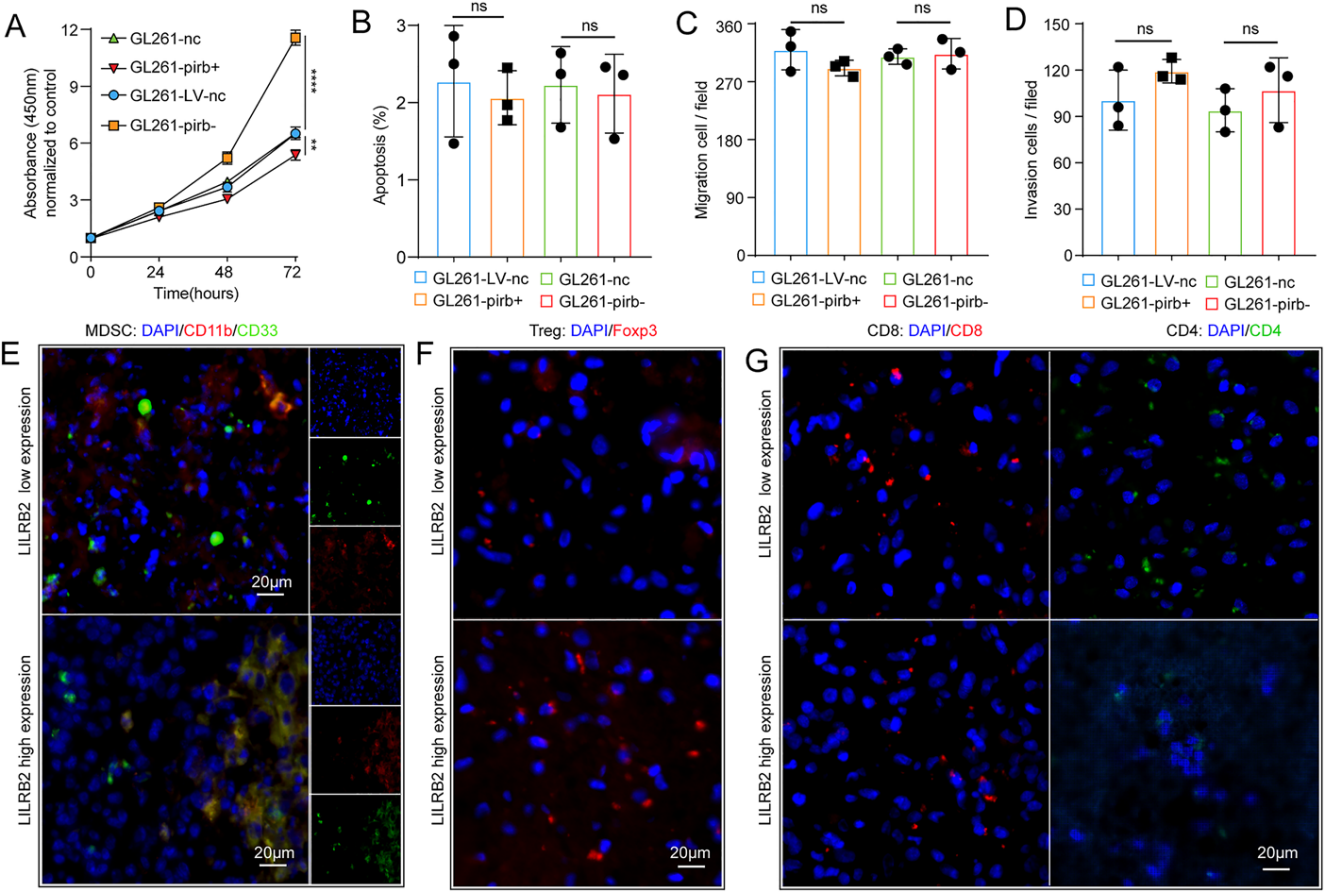
Pirb promotes GBM progression through an immunosuppressive TME. **A** The proliferation of GL261-LV-nc, GL261-pirb^+^, GL261-nc and GL261-pirb^−^ cells detected by CCK8 assays at 24, 48 and 72 h. Statistical analysis of cell apoptosis **B**, migration **C** and invasion **D** in GL261-LV-nc, GL261-pirb^+^, GL261-nc and GL261-pirb^−^ cells. The presence of MDSCs **E**, Tregs **F**, CD4+ and CD8+ T cells **G** in human GBM tissue detected by IF. *P < 0.05, ** P < 0.01, ***P < 0.001, or ****P < 0.0001, ns indicates no statistical significance

**Fig. 3:**
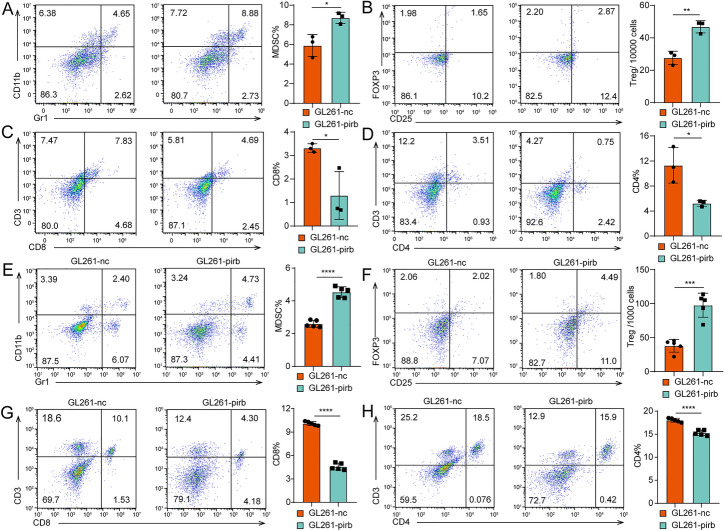
High pirb expression leads to immunosuppression. The percentages of MDSCs **A**, Tregs **B**, CD8+ T cells **C** and CD4+ cells **D** in the tumor sites detected by flow cytometry. The percentages of CD8+ T cells **E**, MDSCs **F**, CD4+ T cells **G** and Treg cells **H** in the spleens of tumor-bearing mice detected by flow cytometry. *P < 0.05, ** P < 0.01, ***P < 0.001, or ****P < 0.0001, ns indicates no statistical significance

The original article has been corrected.

